# Diagnostic Value and Prognostic Use of Presepsin Versus Procalcitonin in Sepsis

**DOI:** 10.7759/cureus.5151

**Published:** 2019-07-16

**Authors:** Deepak P Venugopalan, Gopalakrishna Pillai, Sajitha Krishnan

**Affiliations:** 1 Internal Medicine, Aster Medcity, Kochi, IND; 2 Internal Medicine, Amrita Institute of Medical Sciences and Research Centre, Kochi, IND

**Keywords:** sepsis, procalcitonin, presepsin

## Abstract

Background: Sepsis is a medical problem beyond belief despite the use of modern antibiotics and frequently updated guidelines on resuscitation therapies. It is at this crucial juncture where inflammatory biomarkers have been providing an incredible and easy way to detect sepsis. Various biomarkers have been used over the past decades in medicine to evaluate sepsis, each one better than the previous and having its own pros and cons. In this study, we assess the role of presepsin, which is a CD-14 polypeptide, and procalcitonin which has for some time been the inflammatory marker of choice in sepsis. This is the first study of presepsin as a sepsis biomarker in Indian adults.

Methods: A prospective observational study was conducted in 48 patients who were diagnosed to have sepsis either on admission to the hospital or during their stay in hospital according to the American College of Chest Physicians/Society of Critical Care Medicine (ACCP/SCCM) guidelines during the period from October 2015 to January 2017, after fulfilling all inclusion and exclusion criteria. Efficacy of both inflammatory markers was studied from blood drawn from the patient at the same time, with the same prick.

Results: A total of 48 patients were included in this study. The superiority of presepsin over procalcitonin was evident with presepsin having a sensitivity of 46.2 and specificity of 100 and procalcitonin having a sensitivity of 46.2 and specificity of 31.8. The P value of the presepsin results was significant at <0.001. Along with it, presepsin also proved to be a very reliable marker for 28-day mortality with all 12 patients in the presepsin positive group expiring (P value: <0.001). Receiver operating characteristic (ROC) curve was plotted to try and define an optimal normal value for presepsin in an Indian population and the value calculated was 93.71 with a sensitivity of 65.4 and a specificity of 68.2.

Conclusion: This study shows the superiority of presepsin over procalcitonin as it has much better specificity and a similar sensitivity than procalcitonin and is a better indicator of 28-day mortality. The new cut off that we have postulated here for presepsin improves the efficiency of the inflammatory marker by increasing its sensitivity at the cost of decreasing its specificity slightly.

## Introduction

Sepsis is a medical problem beyond belief. It is still the bane that haunts the entirety of medical specialty in some way or the other. Surgeons fear it in their patients pre-operatively and especially post operatively. Physicians fight it on a daily basis and para medical specialties try and find answers to questions posed by sepsis throughout their careers. It is one of the biggest causes of morbidity and mortality in critically ill patients. All this is despite the use of modern antibiotics and frequently updated guidelines on resuscitation therapies. The biggest problem with sepsis is the difficulty in obtaining an early diagnosis and therefore timely intervention. The moment a patient comes into a hospital and sepsis is suspected by the doctor in charge, there is no single test that can tell us for sure if the patient actually has sepsis or if he is suffering from an illness that is masquerading as sepsis such as a malignancy or non-infective fever.

The already complicated world of sepsis and its management is further rendered difficult by the presence of the large variety of organisms that can cause sepsis. Anything from bacteria to viruses, fungi, and protozoa can cause sepsis in human beings. The treatment for all of these agents are not the same and the specific drugs needed only encapsulates the wide spectrum that we are dealing with in sepsis. The good news is that while all the above-listed organisms can cause sepsis, the majority of infections are due to bacterial invasions. Hence, if we can have a tool which can accurately predict the presence or absence of bacterial sepsis, it will enable the global fraternity to deal with the nuisance of sepsis in a much more effective manner.

It is at this crucial juncture where inflammatory biomarkers have been providing an incredible and easy way to detect sepsis. Recent interest has focused on these newer biomarkers for early diagnosis, risk stratification, and ultimately the evaluation and prognosis of sepsis. Various biomarkers have been used over the past decades in medicine to evaluate sepsis, each one better than the previous and having its own pros and cons.

Although procalcitonin was better than the earlier used inflammatory markers, the reliability was still a problem that was plaguing the widespread use of procalcitonin. The other issue with procalcitonin was that the sensitivity of procalcitonin as studied in a meta-analysis, [1] although higher than the previously mentioned markers was found to be 76% and a specificity of 70%. This showed that there was significant room for improvement and the need for improvement was also clinically important and would aid patients suffering from sepsis.

It is at this juncture that presepsin has been postulated as a newer generation inflammatory marker with a better sensitivity and specificity. It is a CD14 polypeptide, the high-affinity receptor for lipopolysaccharide/lipopolysaccharide-binding protein complexes, is a glycoprotein expressed in macrophage, monocyte, and granulocyte cells and their cell membranes. The lipopolysaccharide-binding protein-CD14 complex is released into circulation by shedding of CD14 from the cell membrane, yielding soluble CD14. Presepsin (soluble CD14-ST), a novel biomarker for diagnosing sepsis, is a subtype of soluble CD14, and is a 13 kDa protein that is a truncated N-terminal fragment of CD14 [2].

Presepsin has been proven to be a very good inflammatory marker for sepsis in numerous studies done worldwide. A meta-analysis done by Zhang et al. [3] showed that it had better sensitivity, specificity, and diagnostic accuracy than procalcitonin.

The need of the hour is to have an inflammatory marker which quickly and efficiently predicts sepsis so that adequate and targeted treatment can be initiated at the earliest. The potential of saving millions of lives is something that needs the medical fraternity’s undivided attention.

There have been no prior studies observing the relationship between presepsin and sepsis in an Indian adult population. This study aims to be the first in the country to define this relationship and contemplate the potential of using presepsin as sepsis biomarker on a widespread basis throughout the country.

The primary objective is to assess the diagnostic value and prognostic use of presepsin versus procalcitonin in sepsis. The secondary objective is to possibly establish presepsin as the investigation of choice in sepsis by proving it to be more sensitive and more specific than other available inflammatory markers at present.

## Materials and methods

Selection and description of participants

Study Setting

This study was carried out over a two-year period from 2015-2017 at Amrita Institute of Medical Sciences and Research Centre, Kochi, Kerala, India under the guidance of the Department of Internal Medicine.

Patient Selection

A subset of patients with sepsis were selected from the intensive care unit (ICU) and the emergency room at random. In total, 48 patients in different stages of sepsis were selected to be part of the study. The sample size was calculated based on the results of a previous study done by Liu et al. in 2013 [4]. With a specificity of 63.6%, a sensitivity of 85.7%, a confidence interval of 99%, and an allowable error of 20%, minimum sample size comes to 40. Hence, a minimum of 40 patients were decided to be taken for the study but was further expanded to include 48 patients due to the availability of samples.

Inclusion Criteria

Minimum age requirement for the study was 18 years. There was no maximum age limit set. All patients had clinical features suggestive of sepsis as defined by American College of Chest Physicians/Society of Critical Care Medicine (ACCP/SCCM) [5] at the time of sample collection and were enrolled into the study. Only patients in whom the treating physician ordered Serum Procalcitonin were included in this study.

Exclusion Criteria

Patients were excluded from this study if they were <18 years old. Other exclusions were patients with terminal stage of disease (malignant cancer of any type, acquired immunodeficiency syndrome, end-stage liver or renal disease) and patient/relatives who did not consent to inclusion.

Technical information

Methods of Data Collection

Once a patient was identified as having sepsis according to the ACCP/SCCM definition of the same, the patient's history and characteristics were entered as per the proforma with special attention being focused on the patient’s vitals and sepsis parameters. After the patients were identified as having sepsis, they were further scrutinised by narrowing them down to usage of serum procalcitonin. Only patients in whom serum procalcitonin was ordered by the treating physician as part of the treatment, were included in this study.

After the patients were identified and informed consent was taken, blood was taken from the patient for the purpose of doing serum procalcitonin. The test for presepsin was done from the same sample and no extra needle pricks or sampling was done for the purpose of the study. Concurrently, as the patients were enrolled in the study, their cultures as sent by the treating physician were also followed up. All sent cultures including blood, urine, bronchoalveolar lavage (BAL), sputum or any other bodily fluid was screened in order to check the presence of any possible bacteremia. 

Methods

As presepsin is not a test that is routinely done at the Amrita Institute of Medical Sciences, an enzyme-linked immunosorbent assay (ELISA) kit was imported into the institution via Abexxa Biologics, UK. The ELISA kit came with 96 wells and although that meant that 96 samples were theoretically possible, many wells were lost to standardisation and controls. The samples were collected and stored at -30 degree Celsius in the biochemistry laboratory and results were analysed using the ELISA kit in two different batches. All details of the patient were collected as mentioned in the proforma and were recorded until the required sample size was reached at which time data analysis was carried out in conjunction with the statistician. The normal value used to measure presepsin was 200 mg/dl and for procalcitonin 0.5 mg/dl.

Statistics

McNemar test was used to compare the procalcitonin status and presepsin status with culture positivity or sepsis. Chi-square test was used to compare the procalcitonin levels and presepsin levels with 28-day mortality status. Independent sample t-test was used to compare the procalcitonin and presepsin value among the alive and expired group. Receiver operating characteristic (ROC) curve estimation was used to define a new cut off point for presepsin value in culture-positive and negative cases. Statistical analysis was done using IBM SPSS Statistics 20 Windows (SPSS Inc., Chicago, USA).

## Results

In this study of 48 patients, there were 26 patients who had a culture positivity, thereby representing a percentage of 54.1% of patients taken as part of the study. Concurrently, there were 22 patients who did not have any sort of culture positivity during their stay in the hospital at the time of the study being conducted. They represented 45.83% of the total study population (Table 1).

Analysis of the data showed that out of the 48 patients included in the study, there were 27 cases in which procalcitonin was positive and 21 cases in which procalcitonin was negative. Further analysis of the same showed that procalcitonin was elevated in 15 patients (55.6%) where there was no culture positivity and it was also elevated in 12 patients (44.4%) in which there was culture positivity (Table 2).

Analysis of the presepsin data collected during this study showed that out of 48 patients included in the study, there were 36 patients in which presepsin was normal and 12 patients in which presepsin was elevated. Further analysis of the data showed that presepsin was elevated in 12 patients (100%) of the culture-positive cases (Table 3). Comparison of the efficiency of presepsin vs. procalcitonin showed presepsin to have the same sensitivity as procalcitonin but a much better specificity, positive predictive value, negative predictive value, and accuracy (Table 4). 

**Table 1:** Primary Table of Enrolled Participants

Table	1: Primary Table of Enrolled participants					
Sex		Males			Females	
Numbers enrolled		25			23
Culture	Number			Percentage		
Positive	26			54.16%		
Negative	22			45.83%		

**Table 2:** Procalcitonin Response to Cultures

Procalcitonin	Culture Positivity		P value
	No	Yes	
	n (%)	n (%)	
<=0.5	7 (33.3)	14 (66.7)	1.000
>0.5	15 (55.6)	12 (44.4)	

**Table 3:** Presepsin Response to Sepsis

Presepsin	Culture Positivity		P value
	No	Yes	
	n (%)	n (%)	
<=200	22 (61.1)	14 (38.9)	<0.001
>200	--	12 (100.0)	

**Table 4:** Comparison of efficiency of Procalcitonin Versus Presepsin

	PROCALCITONIN	PRESEPSIN
Sensitivity	46.2	46.2
Specificity	31.8	100
Positive Predictive Value	44.4	100
Negative Predictive Value	33.3	61.1
Accuracy	39.6	70.8

28-day mortality comparison of procalcitonin versus presepsin

The 28-day mortality of patients in either groups was also analysed and it was found that there were 11 patients (52.4%) in the procalcitonin negative group who survived at the end of 28 days. There were also 10 patients (47.6%) in the procalcitonin negative group who expired at the end of 28 days (Table 5).

**Table 5:** Comparison of 28-day Mortality in Presepsin Versus Procalcitonin SD: Standard deviation.

	28 day mortality	N	Mean	SD	P Value
PROCALCITONIN	Alive	30	3.6	7.1	0.342
	Expired	18	9.7	26.0	
PRESEPSIN	Alive	30	79.7	50.2	0.001
	Expired	18	432.3	387.3	

Concurrently, it was also found that there were 19 patients (70.4%) in the procalcitonin positive group who survived at the end of 28 days. There were also eight patients (29.6%) in the procalcitonin positive group who expired at the end of 28 days. The 28-day mortality of patients in either groups was also analysed and it was found that there were 30 patients (83.3%) in the presepsin negative group who survived at the end of 28 days. There were also six patients (16.7%) in the presepsin negative group who expired at the end of 28 days. Concurrently, it was also found that all 12 patients (100%) in the presepsin positive group expired at the end of 28 days. The P value of the same was significant with a value of <0.001

ROC curve to calculate the normal value of presepsin in Indian population

**Figure 1:**
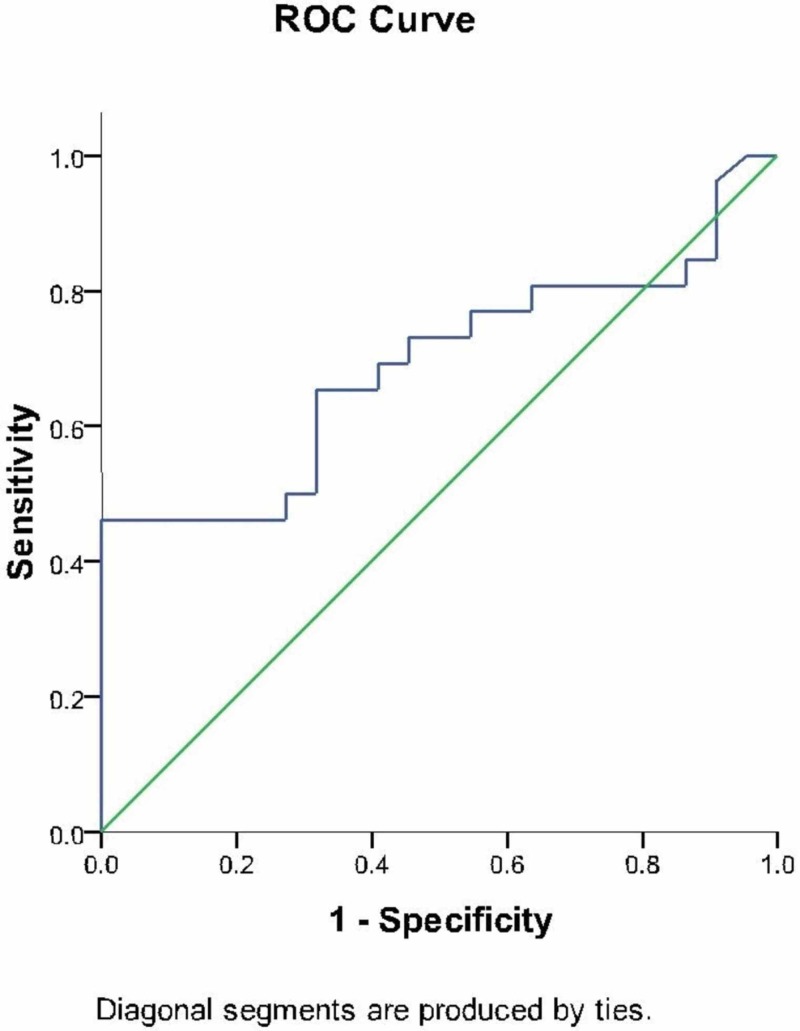
Receiver Operating Characteristic (ROC) Curve for Presepsin

As with the coordinates of the ROC curve which is given in Figure 1, the presepsin value enabling the best sensitivity and specificity is 93.7100. The sensitivity at this value is .654 and 1- specificity is .318. The AUROC (area under ROC) is 0.688 and is a satisfactory indicator of the significance of the study. Analysis of the data with the new value of 93.7 applied as normal, there were 24 patients with presepsin negative and 24 patients with presepsin positive. With a value of 93.71, the analysis of data meant that there were 15 patients (62.5%) in the presepsin negative and culture-negative group and nine patients (37.5%) in the presepsin negative and culture-negative group (Table 6).

Concurrently, there were seven patients (29.2%) in the presepsin positive and culture-negative group. There were also 17 patients (70.8%) in the presepsin positive and culture-positive group. The P value with this was 0.804. The positive predictive value with a value of 93.71 is 70.8, along with a negative predictive value of 62.5 and an accuracy of 66.7. The sensitivity here is 65.4 and the specificity is 68.2 (Table 7).

28-day mortality of presepsin with a normal value of 93.7100: With a value of presepsin set at 93.7100, 21 patients (87.5%) remained alive in the presepsin negative group at the end of 28 days. There were three patients (12.5%) who expired at the end of 28 days in the presepsin negative group. Concurrently, there were nine patients (37.5%) who remained alive in the presepsin positive group at the end of 28 days. Also, there were 15 patients (62.5%) who expired in the presepsin positive group. The P value in this analysis remained significant at 0.001 (Table 8).

**Table 6:** Presepsin Response to Sepsis With a Normal Value of 93.7100

Presepsin	Culture Positivity		P value
	No n (%)	Yes n (%)	
<=93.7	15 (62.5)	9 (37.5)	0.804
>93.7	7 (29.2)	17 (70.8)	

**Table 7:** Efficiency of Presepsin in Sepsis With a Normal Value of 93.7100

PARAMETER OF EFFICIENCY	VALUE
Sensitivity	65.4
Specificity	68.2
Positive Predictive Value (PPV)	70.8
Negative Predictive Value (NPV)	62.5
Accuracy	66.7

**Table 8:** 28-day Mortality Prediction of Presepsin With a Value of 93.7100

Presepsin	28-day Mortality		P value
	Alive n (%)	Expired n (%)	
<=93.7	21 (87.5)	3 (12.5)	0.001
>93.7	9 (37.5)	15 (62.5)	

## Discussion

The purpose of this study was to assess the diagnostic value and prognostic use of presepsin vs. procalcitonin in sepsis. The reliability of either inflammatory markers was studied to further study the role of inflammatory markers in sepsis and to arm physicians with a tool to fight sepsis better. This is the first study of its kind to be done in India as the role of presepsin in sepsis in an adult population has not been studied before in this country. Various studies done in countries outside of India have shown presepsin to have better sensitivity and specificity than procalcitonin.

The study spanned from 2015-2017 and was conducted at Amrita Institute of Medical Sciences, Kochi under the guidance of the Department of General Medicine. Patients who were suspected of having sepsis were included in the study. Sepsis was confirmed by ACCP/SCCM [4] criteria for sepsis. Then, these patient’s cultures (blood, urine, BAL, sputum, pus or any other relevant culture) were compared to their corresponding procalcitonin and presepsin values and the results were analysed to evaluate their diagnostic superiority. The prognostic value of both the inflammatory markers in question were evaluated by comparing their respective 28-day mortality rates.

In this study, there were a total of 48 patients enrolled who qualified the pre-requisite of having sepsis according to the ACCP/SCCM criteria for sepsis. Out of these 48 patients, there were 26 patients (54.1%) who had a positive culture and there were 22 patients (45.8%) who had a negative culture. The sample size was randomly selected but ended up having a mild majority of patients with a positive culture. The demographics of the study population showed that there were 25 males and 23 females in the study.

Analysis of procalcitonin

More in-depth analysis of the data, focusing on the procalcitonin part showed that out of the 48 patients included in the study, there were 27 cases in which procalcitonin was positive and 21 cases in which procalcitonin was negative. Further analysis of the same showed that procalcitonin was elevated in 15 patients (55.6%) where there was no culture positivity and it was also elevated in 12 patients (44.4%) in which there was culture positivity. Concurrently, it was also noticed that procalcitonin remained normal in 7 patients (33.3%) where there was no culture positivity. It also remained normal in 14 patients (66.7%) in whom there was culture positivity recorded during the time of the study. This meant that with a normal value of procalcitonin set at 0.5mg/dl, it had a positive predictive value of 44.4 and a negative predictive value of 33.3 along with an accuracy of 39.6. It also meant that the sensitivity of procalcitonin was 46.2 and the specificity was 31.8.

The interpretation of the procalcitonin data shows that although the sensitivity of the test was just average, the specificity of the test was incredibly poor. This suggests that procalcitonin as an inflammatory marker has very poor reliability in the field of sepsis and does not co-relate to clinical significance and has a poor P value of 1.00.

Analysis of presepsin

The presepsin data meant that there were no patients who were found to have a presepsin positivity in the culture-negative group. Concurrently, it also meant that there were 22 patients (61.1%) who had normal presepsin values in the group of patients without culture positivity and there were 14 patients (38.9%) who had normal presepsin values in the group of patients with culture positivity. This meant that presepsin values had a significant P value of <0.001. The above values meant that presepsin had a positive predictive value of 100, a negative predictive value of 61.1 and an accuracy of 70.8. It also meant that presepsin had a sensitivity of 46.2 and a specificity of 100. With the above-mentioned values, it is abundantly clear the superiority of presepsin with respect to procalcitonin. The specificity of presepsin is the best possible result, although the sensitivity remains average. The significant P value of <0.001 further backs up this claim. Various studies [3] done abroad show similar superiority of presepsin over procalcitonin.

28-day mortality prediction of procalcitonin vs. presepsin

Procalcitonin Analysis

The 28-day mortality of patients in either groups was also analysed and it was found that there were 11 patients (52.4%) in the procalcitonin negative group who survived at the end of 28 days. There were also 10 patients (47.6%) in the procalcitonin negative group who expired at the end of 28 days. Concurrently, it was also found that there were 19 patients (70.4%) in the procalcitonin positive group who survived at the end of 28 days. There were also 8 patients (29.6%) in the procalcitonin positive group who expired at the end of 28 days.

Presepsin Analysis

The 28-day mortality of patients the presepsin group was also analysed and it was found that there were 30 patients (83.3%) in the presepsin negative group who survived at the end of 28 days. There were also 6 patients (16.7%) in the presepsin negative group who expired at the end of 28 days. Concurrently, it was also found that all 12 patients (100%) in the presepsin positive group expired at the end of 28 days. The P value of the same was significant with a value of <0.001. With this, it is again quite clear that presepsin is a much better predictor of 28-day mortality than procalcitonin. The fact that all patients in the presepsin positive group expired at the end of 28 days shows the incredibly adverse effect it has on mortality.

Defining a New Normal Value for Presepsin

As presepsin is a relatively new inflammatory marker and its variation in different demographics has not been studied in detail yet and also because of the high specificity and relatively low sensitivity of presepsin in this study, we tried to define a better cut off limit for presepsin in an Indian population by plotting a ROC curve. This being one of the first studies in India on presepsin further requires validation in a larger study group but we hope to define a cut off for further studies with this ROC curve. As with the coordinates of the ROC curve, the presepsin value enabling the best sensitivity and specificity is 93.7100. The sensitivity at this value is .654 and 1- specificity is .318. The AUROC is 0.688 and is a satisfactory indicator of the significance of the study.

Analysis of the Data with the New Value of 93.7100 Applied as Normal

After taking the newly predicted normal value of 93.71, there were 24 patients with presepsin negative and 24 patients with presepsin positive. It gave an equal number of patients in either category to base our study on. With a value of 93.71, the analysis of data meant that there were 15 patients (62.5%) in the presepsin negative and culture-negative group and nine patients (37.5%) in the presepsin negative and culture-negative group. Concurrently, there were seven patients (29.2%) in the presepsin positive and culture-negative group. There were also 17 patients (70.8%) in the presepsin positive and culture-positive group. The P value with this was 0.804. The positive predictive value with a value of 93.71 is 70.8, along with a negative predictive value of 62.5 and an accuracy of 66.7. The sensitivity here is 65.4 and the specificity is 68.2. This analysis, after setting a postulated new normal value of 93.7100 shows that the sensitivity of presepsin increases significantly but the specificity decreases. This is in order to achieve the best possible sensitivity and specificity. As sensitivity is more of an essential parameter than specificity in sepsis, it is a sacrifice worth making in order to improve the overall efficiency of presepsin. The 28-day mortality was also significant (P value of 0.001) when it was recalculated with the value of 93.7100.

Strengths and Limitations

This study is one of the first studies in India to study presepsin in an Indian population. It lays a marker for further studies in this field. Also, by defining a normal limit for presepsin in the Indian population, this study aims to facilitate widespread research and clinical use of presepsin which would benefit physicians on a daily basis. This study also studied a direct comparison between presepsin and procalcitonin in sepsis at the same time period as the sample for both the tests were drawn together. The limitations of the study include that the serial rise of presepsin and procalcitonin during a patient’s stay in the hospital during the treatment for sepsis has not been studied in this study and prognostication of presepsin and procalcitonin has been studied in respect to 28-day mortality. Although cultures are probably the gold standard to confirm the presence of an infection in the body, it does not always yield accurate results. Previous treatment with antibiotics, occult infections and the organism involved in sepsis can all adversely affect the result of cultures. Multiple patients have co-existing infections and although cultures will most likely define these infections, it is still possible that different organisms co-existed in many patients thereby reducing the validity of the cultures.

## Conclusions

The conclusions that can be derived include the fact that this study shows the superiority of presepsin over procalcitonin as it has much better specificity and a similar sensitivity than procalcitonin. The 28-day mortality of presepsin points to the fact that it is also a significant marker of mortality in sepsis. The new cut off that we have postulated here for presepsin improves the efficiency of the inflammatory marker by increasing its sensitivity at the cost of decreasing its specificity slightly. These factors can be used to make decisions in management, including the need for ICU stabilisation, by the admitting physician. Aggressive treatment of those patients who are found to have a high presepsin value can potentially save lives and improve mortality in sepsis by a very significant margin.
